# Supplementary material for: Collaborative intelligence in AI: Evaluating the performance of a council of AIs on the USMLE
Source: PLOS Digit Health. 2025 Oct 9;4(10):e0000787. doi: 10.1371/journal.pdig.0000787 (PMC12510544; doi:10.1371/journal.pdig.0000787)
Supplement: S2 File — (ZIP) [file pdig.0000787.s002.zip › S2 Data Raw data files containing unprocessed question inputs and LLM outputs including transcripts of deliberations/STEP3_Question-100-1_Type-MC-NJ.docx]

**Facilitator** **[Round 1]:** A 57-year-old man was admitted to the intensive care unit from the emergency department 3 hours ago for treatment of an acute exacerbation of chronic obstructive pulmonary disease. He was brought to the emergency department by ambulance following a 2-hour history of severe shortness of breath and non-productive cough. Chest x-ray obtained on arrival showed significant air-trapping but no infiltrate. The patient was intubated in the emergency department, but now his ventilator pressure alarm is sounding and his blood pressure is dropping. Medical history is otherwise unremarkable. Medications include ipratropium and prednisone. He has smoked 11⁄2 packs of cigarettes daily for about 40 years. Vital signs are temperature 37.7°C (99.9°F), pulse 106/min, respirations 12/min on intermittent mandatory ventilation, and blood pressure 72/46 mm Hg. Pulse oximetry on 40% FIO₂ shows an oxygen saturation of 91%. He appears obtunded and is poorly responsive. Physical examination shows jugular venous distention in the supine position; trachea is midline. Auscultation of the lungs discloses decreased breath sounds bilaterally with inspiratory and expiratory wheezing and a prolonged expiratory phase. Examination of the upper extremities shows 2+ digital clubbing bilaterally. There is no cyanosis or edema. Which of the following is the most appropriate management? (A) Adjustment of the ventilator settings (B) Insertion of bilateral chest tubes (C) Intravenous dopamine (D) Intravenous low-molecular-weight heparin (E) 1-L bolus of intravenous 0.9% saline (F) Needle decompression

**AI_E:** RESTATING THE QUESTION: This clinical scenario presents a 57-year-old male with a history of heavy smoking, currently in the intensive care unit following an acute exacerbation of his chronic obstructive pulmonary disease (COPD). Although he was intubated and put on a ventilator upon his arrival, an alarm on the device is now sounding, his blood pressure is falling, and he appears less alert. Additionally, the patient exhibits signs of jugular venous distention and decreased breath sounds, amongst other symptoms. The question concerns which course of therapeutic action we should take from the given options. PLANNING: Several assumptions can potentially underlie this scenario. The ventilator alarm could be sounding due to inadequate settings or lung disease deterioration, causing increased airway resistance or decreased lung compliance. Prolonged expiratory wheezing and jugular vein distension might indicate dynamic hyperinflation (breath stacking) and tension pneumothorax. Falling blood pressure may also suggest either a decreased intravascular volume or cardiac output. Existing factors such as the patient's COPD, smoking history, or even a new medical condition, could be influencing the situation. The assumptions leading to dynamic hyperinflation due to severe COPD exacerbation appear to be more appropriate for clinical prioritization. CLINICAL REASONING: In a patient with severe COPD, the difficulty of exhaling air contributes to dynamic air trapping (also known as auto-positive end expiratory pressure - auto PEEP or breath-stacking). This happens when the patient starts the next inspiration before finishing the previous expiration. If the patient attempts to inhale before fully exhaling, the air from the previous breath is essentially "stacked" onto the air from the current breath—leading to significant lung hyperinflation. This hyperinflation can shift the mediastinum, impeding venous return and generating hemodynamic instability. This phenomenon might explain the ventilator pressure alarm activation, decreased blood pressure, obtunded status (due to decreased cerebral perfusion), and jugular venous distension (from impaired venous return). CONCLUDING: Considering the clinical reasoning: the synchrony between the patient and the ventilator is disrupted by dynamic hyperinflation, commonly seen in diseases with excessive airflow obstruction such as severe COPD. This problem when unnoticed, can lead to cardiovascular compromise due to increased intrathoracic pressures. Hence, appropriate management would address this ventilation dyssynchrony to prevent the risk of "breath stacking". RESPONSE: The most appropriate management in this clinical scenario is (A) an adjustment of the ventilator settings to alleviate the dyssynchrony and avoid the effects of dynamic hyperinflation.

**AI_A:** RESTATING THE QUESTION: A 57-year-old patient, being treated in the intensive care unit for an acute exacerbation of chronic obstructive pulmonary disease (COPD), is seeing worsening health indicators. Despite being intubated, his ventilator pressure alarm is sounding, and his blood pressure is dropping. The patient has been on medications like ipratropium and prednisone and has a long history of tobacco use. Current vital signs include a slightly elevated temperature, a high pulse rate, low blood pressure, and an oxygen saturation of 91% on 40% FIO2. His state is described as obtunded or severely drowsy. Physical examination shows evident lung issues and digital clubbing. Among the given options for intervention, we must determine the best. PLANNING: The options for management include adjusting the ventilator settings, inserting bilateral chest tubes, administering intravenous dopamine, giving intravenous low-molecular-weight heparin, providing a 1-L bolus of intravenous 0.9% saline, or performing needle decompression. The key information we're working with are the symptoms indicating serious pulmonary distress and possible ventilation issues due to COPD exacerbation and the sounding ventilator alarm suggesting high airway pressures. CLINICAL REASONING: In the case of our patient, the dropping blood pressure and the sounding ventilator pressure alarm signal a ventilation problem that could be due to a myriad of issues. With the patient's prior diagnosis of COPD, one such issue could be dynamic hyperinflation of the lungs, more commonly called "breath stacking." Breath stacking results from inadequate exhalation between breaths and can lead to barotrauma, hypotension, and inefficient ventilation. This complication is especially prevalent in patients with obstructive lung disease, like our patient. The tumultuous combination of a high respiratory rate and a prolonged expiratory phase, as seen in COPD patients, is a common recipe for breath stacking. CONCLUDING: In addressing breath stacking, one plausible option is to alleviate the stress on the lungs by adjusting the ventilator settings. Adjustments might include decreasing the respiratory rate to allow for more time for expiration, or decreasing the inspiratory flow rate or tidal volume. Such adjustments could relieve the intra-thoracic pressure caused by stacked breaths, potentially normalizing the patient's blood pressure and improving his ventilation condition. RESPONSE: Therefore, following the key aspects of clinical reasoning and understanding of COPD pathophysiology, the most appropriate management for the patient in this scenario seems to be the adjustment of the ventilator settings (option A).

**AI_D:** RESTATING THE QUESTION: The query asks for an appropriate line of management for a 57-year-old patient hospitalized with acute exacerbation of chronic obstructive pulmonary disease (COPD). He's experiencing severe symptoms that have led to intubation and is currently unstable due to a sounding ventilator pressure alarm and dropping blood pressure. The patient's clinical picture suggests the possibility of mechanical ventilation complications, and therefore, we need to tread with caution and choose an appropriate treatment strategy. PLANNING: The assumptions that we can make from the given clinical data are that the patient's condition is likely due to complications related to COPD and its treatment, particularly mechanical ventilation, or progression of the disease process. Evaluating the options, we can rule out bacterial pneumonia as there's no infiltrate on chest x-ray. Pulmonary embolism seems unlikely due to the absence of associated symptoms or risk factors. Intravenous dopamine is used in shock states but wouldn't necessarily address the etiology of the patient's condition. Intravenous low-molecular-weight heparin is used for preventing coagulopathy in bed-bound patients but again doesn't directly address the patient's current condition. A 1-L bolus of 0.9% saline might worsen the patient's pulmonary status. Needle decompression, typically used for tension pneumothorax, doesn't seem applicable here as the patient's physical examination does not suggest tension pneumothorax. Insertion of bilateral chest tubes could be considered if there was a large pleural effusion, but chest x-ray only demonstrated air-trapping without indication of effusion. CLINICAL REASONING: Connecting these pieces of information, we need to focus on finding an option that addresses the potential mechanical ventilation complications or a worsening clinical status related to COPD. If we work under the assumption that the ventilator pressure alarm and a dropping blood pressure may indicate a complication associated with mechanical ventilation, such as dynamic hyperinflation (possible because of the air trapping seen in the x-ray and the patient's COPD condition), we can hypothesize that adjusting the ventilator settings could address this issue. CONCLUDING: Step by step, we started from our assumption of a mechanical ventilation issue which was supported by evidence of air-trapping in a COPD patient. By reasoning through the array of possible conditions that could arise from this situation, we arrive at the conclusion that this might be a case of dynamic hyperinflation. As such, it's feasible that adjusting the ventilator settings could alleviate this problem and improve the patient's status. RESPONSE: Therefore, the most appropriate management in this case would be (A) Adjustment of the ventilator settings.

**AI_B:** RESTATING THE QUESTION: The medical staff handling the case of a 57-year-old male patient needs guidance on management options after presenting with acute chronic obstructive pulmonary disease. His symptoms, following a moderate duration of severe shortness of breath and dry cough, alarmingly include current blood pressure drops and ventilator pressure alarm sounds despite him being intubated earlier. His medical history shows no other significant conditions, and he takes ipratropium and prednisone. Other concerning signs include his reduced oxygen saturation, obtunded state, decreased lung sounds, jugular venous distention, and the presence of digital clubbing. The management options being considered range from adjusting ventilator settings, insertion of chest tubes, administering intravenous dopamine or low-molecular-weight heparin or 0.9% saline, or needle decompression. PLANNING: Starting assumptions point out to exacerbation of COPD and subsequent development of acute ventilatory failure being managed currently, but now the deteriorating hemodynamic profile and ventilator alarm point us towards possible barotrauma, which often happens in patients suffering from COPD who are mechanically ventilated. This theory would be tested with a secondary assumption that low blood pressure is not due to hypovolemia which would otherwise need intravenous saline loading. Also, it's less likely to be due to low cardiac output or thromboembolic phenomenon which would prompt dopamine or LMWH administration respectively. Overdosing of sedatives leading to central induced hypotension seems less likely given the examination findings. The alternate assumption, despite no explicit evidence of tension phenomena, would also evaluate the need for emergent needle decompression over a less emergent intervention like ventilator setting adjustments or a more drastic, but rare intervention required in barotrauma, bilateral chest tube insertion. CLINICAL REASONING: With this patient's presentation of obtunded state, low blood pressure, and a ventilator pressure alarm, the most likely explanation is dynamic hyperinflation, also known as breath stacking or auto-PEEP (Positive end-expiratory pressure), a common complication in patients with COPD on mechanical ventilation. Breath stacking occurs when a patient on a ventilator begins the next inspiration before they've fully exhaled. This causes progressive air trapping (already demonstrated in the X-ray), leading to increase intrathoracic pressure that in turn decreases venous return and cardiac output and manifests as hypotension and jugular venous distension. The presence of alarm from the ventilator, decreased breath sounds, and decreasing blood pressure further strengthens this perspective. CONCLUDING: If dynamic hyperinflation is occurring, our main goal is to alter ventilation in a way that allows more time for exhalation to prevent air trapping. That can be achieved by adjusting the ventilator settings. Other interventions like needle decompression, chest tubes, or vasopressors such as dopamine are not appropriate unless there are other clinical indicators. LMWH and IV normal saline do not seem relevant to the patient's presentation. RESPONSE: Given this clinical scenario and working through the reasoning process, the most appropriate management for this patient would be (A) Adjustment of the ventilator settings to prevent further dynamic hyperinflation.

**AI_C:** RESTATING THE QUESTION: A 57-year-old individual came in with an acute episode of chronic obstructive pulmonary disease (COPD) that developed over the course of two hours, characterised by severe breathlessness and a dry cough. His medical history is notable mainly for long-term smoking. The patient seems mentally unresponsive and his physical examination reveals features such as distended jugular veins, decreased breath sounds on both sides of the chest, and digital clubbing of the fingers. Despite intubation and mechanical ventilation, the patient's blood pressure is falling, and the ventilator alarm is sounding, indicating potential issues. Based on these observations, what should be the best next step in clinical management? PLANNING: The first assumption is that the man's symptoms are due to an acute exacerbation of his underlying COPD. This is supported by his smoking history, the presence of digital clubbing, and the identification of significant air trapping on his chest x-ray. However, because his condition is deteriorating even after intubation and ventilation, an alternate assumption is that there is another pathology contributing to his acute deterioration. The pathogenesis of COPD includes air trapping and elasticity loss that leads to end-expiratory lung volumes’ increase which can predispose to pneumothorax. A pneumothorax would underlie his deteriorating clinical status and trigger the ventilator's pressure alarm despite the intervention. Based on this pathophysiological reasoning, a pneumothorax should be clinically prioritized and evaluated first. CLINICAL REASONING: The sharp drop in blood pressure together with the activation of the ventilator alarm points toward possible autonomic dysregulation, like we can see with a tension pneumothorax. In these cases, the trapped air in the pleural space can compress the heart and major vessels, leading to a drop in venous return, thus a lower cardiac output and circumstantially hypotension. Also, the trapped air in the chest can rise intrathoracic pressure and trigger the ventilator's pressure alarm. Tension pneumothorax should be treated immediately to prevent fatal cardiovascular collapse by decompressing the pleural space to restore venous return and prevent further air-trapping in the chest due to the one-way valve mechanism of pneumothorax. CONCLUDING REMARKS: Given the acute worsening of this patient's condition despite maximal respiratory support and the presence of physical findings, a tension pneumothorax should be considered as the potential culprit. This would require immediate decompression to relieve the trapped air and restore normal physiology. RESPONSE: Hence, with respect to the therapeutic options offered, the correct intervention seems to be (F) Needle decompression. This would most directly address the presumed underlying issue here, a tension pneumothorax. It is critical to restore normal pressures within the thoracic cavity to stabilize the patient, improve cardiac output and consequently his blood pressure.

**Facilitator** **[Round 2]:** SUMMARIES: AI_A: The patient's dropping blood pressure and sounding ventilator pressure alarm suggest a ventilation problem, possibly due to "breath stacking" common in COPD patients. The best intervention would be to adjust the ventilator settings to alleviate the stress on the lungs. AI_B: The patient's symptoms suggest dynamic hyperinflation, also known as breath stacking, a common complication in COPD patients on mechanical ventilation. The most appropriate management would be to adjust the ventilator settings to prevent further dynamic hyperinflation. AI_C: The patient's symptoms suggest a possible tension pneumothorax, which would require immediate decompression to relieve the trapped air and restore normal physiology. The best intervention would be needle decompression. AI_D: The patient's symptoms suggest a possible complication associated with mechanical ventilation, such as dynamic hyperinflation. The best intervention would be to adjust the ventilator settings to alleviate this problem and improve the patient's status. AI_E: The patient's symptoms suggest dynamic hyperinflation due to severe COPD exacerbation. The best intervention would be to adjust the ventilator settings to alleviate the dyssynchrony and avoid the effects of dynamic hyperinflation. KEY DIFFERENCES: The key difference in the council members' perspectives lies in their interpretation of the patient's symptoms. While AI_A, AI_B, AI_D, and AI_E all believe that the patient's symptoms suggest dynamic hyperinflation or "breath stacking" and recommend adjusting the ventilator settings, AI_C interprets the symptoms as indicative of a tension pneumothorax and recommends needle decompression. QUESTION TO CLARIFY DISAGREEMENTS: What specific clinical indicators or patient history factors led you to your conclusion about the patient's condition, and why do you believe your recommended intervention is the most appropriate in this case?. Based on your preceding response, how would you answer the original question: [A 57-year-old man was admitted to the intensive care unit from the emergency department 3 hours ago for treatment of an acute exacerbation of chronic obstructive pulmonary disease. He was brought to the emergency department by ambulance following a 2-hour history of severe shortness of breath and non-productive cough. Chest x-ray obtained on arrival showed significant air-trapping but no infiltrate. The patient was intubated in the emergency department, but now his ventilator pressure alarm is sounding and his blood pressure is dropping. Medical history is otherwise unremarkable. Medications include ipratropium and prednisone. He has smoked 11⁄2 packs of cigarettes daily for about 40 years. Vital signs are temperature 37.7°C (99.9°F), pulse 106/min, respirations 12/min on intermittent mandatory ventilation, and blood pressure 72/46 mm Hg. Pulse oximetry on 40% FIO₂ shows an oxygen saturation of 91%. He appears obtunded and is poorly responsive. Physical examination shows jugular venous distention in the supine position; trachea is midline. Auscultation of the lungs discloses decreased breath sounds bilaterally with inspiratory and expiratory wheezing and a prolonged expiratory phase. Examination of the upper extremities shows 2+ digital clubbing bilaterally. There is no cyanosis or edema. Which of the following is the most appropriate management? (A) Adjustment of the ventilator settings (B) Insertion of bilateral chest tubes (C) Intravenous dopamine (D) Intravenous low-molecular-weight heparin (E) 1-L bolus of intravenous 0.9% saline (F) Needle decompression]

**AI_A:** RESPONSE: This patient's clinical findings, such as a high-pressure alarm, generalized wheezing, and hemodynamic instability, are suggestive of dynamic hyperinflation in the setting of acute COPD exacerbation. Mechanically ventilated patients with obstructive lung disease, like COPD, are at risk of dynamic hyperinflation ("breath stacking") due to insufficient time for expiration and gas trapping. This can lead to an increase in intrathoracic pressure, which subsequently decreases venous return and thus cardiac output, causing shock. The absence of unilateral reduced breath sounds or tracheal deviation rules out tension pneumothorax, while the absence of leg swelling or respiratory distress shortly after initiation of anticoagulation rules out pulmonary embolism, both of which could also explain shock and hypoxemia. The most appropriate next step in this patient's management should be adjusting the ventilator settings (Choice A). Specifically, reducing the respiratory rate and/or tidal volume can provide more time for exhalation and minimize air-trapping. Other beneficial modifications can include increasing the flow rate or changing to a decelerating flow. Chest tubes (Choice B) would be necessary in the case of a large pneumothorax, which is not observed here. Dopamine (Choice C) could increase the blood pressure but would not address the underlying issue. Heparin (Choice D) would be beneficial in the case of a suspected pulmonary embolism, for which this patient demonstrates no clinical suspicion. Saline bolus (Choice E) may worsen the patient's respiratory status and isn't indicated without signs of hypovolemia. Needle decompression (Choice F) would be used to treat tension pneumothorax, which is not evident in this case based on physical examination.

**AI_D:** RESTATING THE QUESTION: The scenario presents a 57-year-old gentleman who experiences an exacerbation of his chronic obstructive pulmonary disease (COPD) that has led him to be intubated and placed on a ventilator at the intensive care unit. This patient's prior history of heavy smoking, his clinical manifestation of severe shortness of breath, non-productive cough, and the physical exam findings all point towards this COPD exacerbation. Now, his ventilator pressure alarm is sounding, and his blood pressure is dwindling, suggesting a possible clinical complication. The query is about the most suitable management plan in light of this situation. PLANNING: This situation could be a result of several possibilities. COPD patients frequently experience "dynamic hyperinflation" or "breath stacking," which could induce complications on mechanical ventilation. Alternatively, the patient could have developed a tension pneumothorax due to mechanical ventilation. Lastly, the patient may have a form of shock causing the hypotension, and we might need to address that. CLINICAL REASONING: Given the clinical history and presentation, "dynamic hyperinflation" or "breath stacking" seems highly probable. In such a scenario, air gets trapped within the lungs as a COPD patient tries to inhale another breath before completing his exhalation. With a mechanical ventilator that pushes air in at preset intervals, this situation potentially worsens, leading to hyperinflation. When hyperinflation occurs in the lungs, it increases the intrathoracic pressure, eventually leading to decreased venous return, contributing to hypotension - as seen in this patient. CONCLUDING: Taking stock of the above, adjustment of the ventilator settings would be clinically plausible and should be looked up first. Synchronizing the ventilation to the patient's breathing pattern, allowing for longer exhalation times by lowering the respiratory rate, breath size (tidal volume), or the use of pressure-controlled ventilation if not already in use, could bring about the necessary changes to manage dynamic hyperinflation in this patient. RESPONSE: Connectively, given the patient's overall clinical picture and presentation, the most suitable management approach is likely to be (A) Adjustment of the ventilator settings to address the issue of dynamic hyperinflation, reduce the trapped air, and possibly address the concurrently dropping blood pressure due to decreased venous return.

**AI_E:** (1) RESTATING THE QUESTION: The question seeks to determine the best management approach for a 57-year-old male patient who has been admitted to the ICU due to an acute exacerbation of chronic obstructive pulmonary disease (COPD). After intubation in the emergency department, his ventilator pressure alarm began to sound and his blood pressure started dropping. Some notable characteristics of the patient include severe shortness of breath, non-productive cough, significant air-trapping evident on a chest x-ray, usage of ipratropium and prednisone, a long smoking history, low blood pressure, decreased oxygen saturation, obtunded responsiveness, jugular venous distention, and decreased breath sounds with wheezing and prolonged expiratory phase. The question aims to identify the ideal course of action given these observations. (2) PLANNING: The assumptions here include that the patient's ventilator issues and dropping blood pressure are due to complications related to an acute COPD exacerbation. One such complication that can occur in mechanically ventilated COPD patients is dynamic hyperinflation, also known as breath stacking, where the patient's inability to adequately exhale leads to a buildup of air in the chest. This can increase intrathoracic pressure, leading to a decrease in venous return and subsequently, low blood pressure. Another possible assumption is the development of tension pneumothorax, but it seemingly lacks key clinical signs, such as tracheal deviation or unilateral decreased breath sounds. (3) CLINICAL REASONING: In this case, the alternate assumption of dynamic hyperinflation appears more likely given the clinical picture and the patient’s known COPD status. The ventilator’s pressure alarm, decreased breath sounds, prolonged expiratory phase, and decreased blood pressure all point towards increased intrathoracic pressure caused by air trapping during mechanical ventilation among COPD patients. Jugular venous distention in this scenario could be due to decreased venous return, secondary to the increase in intrathoracic pressure. The lack of unilateral decreased breath sounds or tracheal deviation makes the possibility of tension pneumothorax less likely. (4) CONCLUDING: Given the above clinical reasoning, dynamic hyperinflation secondary to mechanical ventilation among COPD patients seems to be the most probable cause of the patient's prevailing conditions. As such, the appropriate treatment strategy should revolve around resolving this issue. (5) RESPONSE: The most appropriate management in this scenario would be (A) Adjustment of the ventilator settings. This allows to alleviate the dynamic hyperinflation, assisting in the resolution of the exacerbation of the patient's COPD.

**AI_C:** Responses to the question: AI_A: My conclusion about the patient's condition is based on several factors. Firstly, the patient's medical history of COPD and recent exacerbation, the finding of air-trapping on chest x-ray, and the triggering of the ventilator pressure alarm all suggest dynamic hyperinflation, a common complication in COPD patients on mechanical ventilation. This is further supported by the patient's appearance, including decreased breath sounds and prolonged expiratory phase, as well as his hypotension, likely due to decreased venous return from increased intrathoracic pressure. Considering these indications, adjustment of the ventilator settings would be my answer to the original question as it would alleviate air trapping and stabilize the patient's physiology. AI_B: The clinical indicators that led me to my conclusion include the patient's COPD history, the ventilator pressure alarm, and the patient's hypotension. These signs are indicative of dynamic hyperinflation or breath stacking, a condition where the patient is unable to fully exhale before the next breath is initiated causing air trapping. The most appropriate intervention, in this case, would be to adjust the ventilator settings. This would allow more time for exhalation, preventing further dynamic hyperinflation, and thus would be my answer to the original question. AI_C: The patients' clinical indicators such as decreased breath sounds, hypotension, and the sounding of the ventilator pressure alarm, in my assessment, suggest a possible tension pneumothorax. In such a life-threatening situation, immediate decompression to relieve trapped air and restore systemic blood pressure is vital. Therefore, needle decompression would be my response to the original question. AI_D: The patient's ventilator pressure alarm going off, paired with his dropping blood pressure, and history of COPD all suggest a possible complication associated with mechanical ventilation. Dynamic hyperinflation associated with ventilator setting mismatch can be assumed in this case. I believe adjusting the ventilator settings would rectify this issue, improve the patient's current state, and prevent potential ventilator-related injuries; hence, it would be my answer to the original question. AI_E: The clinical indicators such as the patient's history of COPD, the sounding ventilator pressure alarm, the prolonged expiratory phase, and hypotension suggest dynamic hyperinflation due to dyssynchrony between the patient's breathing and the ventilatory aid. Adjusting the ventilator settings should manage this dyssynchrony, hence preventing further effects of dynamic hyperinflation. Therefore, adjusting the ventilator setting is my answer to the original question. Given these responses, the council agrees that the most appropriate management would be adjusting the ventilator settings. This decision is supported by the majority (AI_A, AI_B, AI_D, and AI_E), except for AI_C who anticipated a different problem (tension pneumothorax).

**AI_B:** 1) RESTATING THE QUESTION: Our council is discussing the treatment plan for a 57-year-old male patient who was admitted to the intensive care unit due to an acute exacerbation of chronic obstructive pulmonary disease (COPD) — a lung disease characterized by chronic obstruction of lung airflow. His symptoms include high ventilator pressure, dropping blood pressure, severe shortness of breath, and non-productive cough. His chest x-ray showed significant air trapping without any infiltrates, indicating no signs of infection or consolidation in the lungs. We are tasked with deciding what the most appropriate management is given these symptoms and medical history. 2) PLANNING: Assumptions when deliberating the treatment options could include potential complications related to his COPD and mechanical ventilation, such as dynamic hyperinflation (also known as breath stacking). This happens when the patient starts the next breath before fully exhaling the previous breath, leading to trapped air and increased lung volumes. Alternatively, we could also suspect tension pneumothorax, a life-threatening condition where air accumulates in the pleural space and cannot escape, causing the lungs to collapse and the heart to shift, which may lead to severe hemodynamic compromise. Other assumptions - such as hypovolemia, venous thrombosis, septic shock or inadequate inotropic support - could also be considered but might be less likely to consider due to the clinical picture and history of the patient. The priority, however, should be to evaluate if the patient's blood pressure drop and ventilator alarm are related to a complication from his lung disease or the mechanical ventilation. 3) CLINICAL REASONING: The patient's prolonged history of smoking, decreased breath sounds with wheezing, and the presence of clubbing all suggest Chronic Obstructive Pulmonary Disease (COPD) with potentially emphysematous changes. These signs suggest long-term damage to the lung structures, leading to air trapping and inadequate gas exchange, further exacerbated under stress conditions, such as acute illness. Mechanical ventilation in these patients can cause dynamic hyperinflation or breath-stacking, leading to increased intrathoracic pressure, reduced venous return, and consequent hypotension. While tension pneumothorax could also cause such a scenario, the absence of other signs such as tracheal deviation or severely decreased (or absent) breath sounds makes this less likely. 4) CONCLUDING: Given the clinical picture and the patient's history, dynamic hyperinflation due to mechanical ventilation in a patient with severe COPD appears to be the most likely cause of the presenting problems. An appropriate measure to handle such a scenario would be adequate adjustment of the ventilator settings. This could include decreasing the tidal volume or the respiratory rate, allowing for more time for exhalation and preventing further air trapping. 5) RESPONSE: Therefore, the most appropriate management for this patient would be (A) Adjustment of the ventilator settings. This would relieve the breath stacking and the associated hemodynamic compromise, by allowing adequate exhalation time and preventing further air trapping.

**Facilitator** The consensus among the AI responses is that the most appropriate management for the 57-year-old male patient, who was admitted to the ICU due to an acute exacerbation of chronic obstructive pulmonary disease (COPD), is the adjustment of the ventilator settings. This conclusion is based on the patient's clinical findings, such as a high-pressure alarm, generalized wheezing, and hemodynamic instability, which suggest dynamic hyperinflation in the setting of acute COPD exacerbation. Mechanically ventilated patients with obstructive lung disease, like COPD, are at risk of dynamic hyperinflation ("breath stacking") due to insufficient time for expiration and gas trapping. This can lead to an increase in intrathoracic pressure, which subsequently decreases venous return and thus cardiac output, causing shock. The absence of unilateral reduced breath sounds or tracheal deviation rules out tension pneumothorax, while the absence of leg swelling or respiratory distress shortly after initiation of anticoagulation rules out pulmonary embolism, both of which could also explain shock and hypoxemia. Therefore, reducing the respiratory rate and/or tidal volume can provide more time for exhalation and minimize air-trapping. Other beneficial modifications can include increasing the flow rate or changing to a decelerating flow. This decision is supported by the majority of the AI responses, except for one that anticipated a different problem (tension pneumothorax).
